# Sex differences in digital dating app use and app-mediated sexual and health outcomes: a systematic review and meta-analysis

**DOI:** 10.1093/sexmed/qfag079

**Published:** 2026-08-24

**Authors:** Aysu Yıldız Karaahmet, Fatma Şule Bilgiç, Duygu Dişli Çetinçay

**Affiliations:** Department of Midwifery, Faculty of Health Sciences, Biruni University, Istanbul 34010, Türkiye; Department of Midwifery, Faculty of Health Sciences, Çanakkale Onsekiz Mart University, Çanakkale 17100, Türkiye; Department of Nursing, Faculty of Health Sciences, Haliç University, Istanbul 34545, Türkiye

**Keywords:** dating applications, sex differences, sexual behavior, sexual health, sexually transmitted infections

## Abstract

**Objective:**

This systematic review and meta-analysis aimed to synthesize sex-based differences in dating app use, app-mediated sexual behaviors, and sexual health outcomes using sex-stratified quantitative data from observational studies.

**Methods:**

Conducted in accordance with PRISMA 2020 guidelines and prospectively registered in PROSPERO, this review synthesized sex-disaggregated data from observational studies published between 2008 and 2025.Systematic searches were performed in PubMed/MEDLINE, Web of Science, PsycINFO, Scopus, and the Cochrane Library. Outcomes included prevalence of dating app use, app-mediated casual sexual encounters, sexual function outcomes, sexual orientation distribution, experience of sexual intercourse, online versus offline partner-seeking behaviors, and history of sexually transmitted infections (STIs). Random-effects meta-analyses were conducted, and heterogeneity was assessed using the *I*^2^ statistic.

**Results:**

Twenty-two observational studies were included. Women were significantly less likely than men to report dating app use (odds ratio [OR] = 0.46, 95% confidence interval [CI], 0.28-0.77; *I*^2^ = 97%). Among dating app users, women had lower odds of sexual function impairment (OR = 0.37, 95% CI, 0.20-0.66; *I*^2^ = 88%) and were less likely to report a sexual minority orientation (OR = 0.34, 95% CI, 0.16-0.69; *I*^2^ = 92%). No statistically significant sex-based differences were observed for app-mediated casual sexual encounters, experience of sexual intercourse, history of STIs, or online versus offline partner-seeking behaviors. Substantial heterogeneity was present across all outcomes.

**Conclusion:**

Dating app–related behaviors and outcomes differ by sex and are not sex-neutral. Men report higher engagement in dating app use and sexual risk–related behaviors, whereas women show distinct patterns in sexual function and psychosocial outcomes. These findings support the use of sex-stratified analytical frameworks and sex-sensitive interventions in digital dating contexts.

## Introduction

Beyond an exclusive focus on sexual risk behaviors, an expanding body of research indicates that dating app use is associated with a broad range of sexual, psychological, and relational outcomes. Dating applications have become a central feature of contemporary partner-seeking, particularly among young adults, reshaping how individuals initiate relationships, negotiate intimacy, and engage in sexual activity.^1^ Empirical studies suggest that dating app use is linked not only to patterns of sexual behavior but also to mental health and well-being, underscoring the need for a more comprehensive understanding of their impacts.^2^

Accumulating evidence indicates that dating app use is associated with diverse psychological outcomes, including depressive symptoms, body image concerns, and reduced well-being for some users, while others report increased social connectedness, perceived partner availability, or sexual satisfaction.^2^^,^^3^ These mixed findings suggest that the effects of dating app use are not uniform but vary considerably across individuals and contexts. Such heterogeneity highlights the importance of examining dating app–related outcomes beyond simplistic risk-based frameworks.

Research focusing on sexual behavior has documented associations between dating app use and casual sexual encounters, sexual debut, and patterns of sexual activity, particularly among young adults and college populations.^3^^,^^4^ However, the magnitude and direction of these associations vary substantially across studies. Some investigations report increased engagement in casual or non-committed sexual encounters among dating app users, whereas others find no clear differences when compared with offline partner-seeking contexts.^1^^,^^2^ These inconsistencies underscore the role of contextual and individual-level factors in shaping dating app–mediated sexual behaviors.

Importantly, emerging evidence suggests that sex differences play a critical role in dating app use and its associated outcomes. Studies have reported sex-specific patterns in motivations for app use, engagement in casual sexual encounters, sexual health outcomes, and experiences of psychological well-being.^2^^,^^3^ In addition, research examining sexual minority populations indicates that dating apps may function differently across sex and sexual orientation groups, particularly with respect to sexual health and psychosocial outcomes.^2^^,^^3^ Despite this, findings related to sex-based differences are often reported as secondary analyses and remain fragmented across individual studies.

Methodological and conceptual heterogeneity further complicates interpretation of the existing literature. Studies vary widely in how dating app use is operationalized, ranging from general exposure to specific behaviors such as app-mediated casual sex or online versus offline partner-seeking. Outcome measures span sexual behaviors, sexual health indicators, psychological well-being, and relational experiences, assessed using diverse instruments and analytic strategies.^2^^,^^3^ Although many studies report sex-stratified results, these data have not been systematically synthesized across outcome domains, limiting the ability to draw robust conclusions about sex-based differences in dating app–related behaviors and outcomes.

Although several recent systematic reviews have synthesized evidence on dating app use in relation to psychological health and well-being, body image, psychosocial correlates, problematic use, and sexually transmitted infections (STIs), these reviews have primarily addressed specific outcomes or selected populations. For example, Sharabi et al. synthesized evidence on psychological health and well-being,^3^ Bowman et al. focused on body image, mental health, and well-being,^2^ Wang et al. examined STIs among men who have sex with men,^5^ Castro and Barrada reviewed the psychosocial correlates of dating app use,^6^ Bonilla-Zorita et al. evaluated problematic dating app use,^7^ and Queiroz et al. systematically reviewed sexual behaviors and STIs associated with dating app use.^8^ However, these reviews did not comprehensively synthesize sex-stratified quantitative evidence across multiple behavioral and sexual health outcomes. To our knowledge, no previous systematic review and meta-analysis has comprehensively evaluated sex-based differences across these outcome domains. Consequently, substantial gaps remain in understanding how dating app use, sexual behaviors, and associated health outcomes differ between women and men across diverse contexts. The present review addresses this gap by quantitatively comparing women and men across multiple behavioral and sexual health outcome domains.

## Objectives and research questions

The aim of this systematic review and meta-analysis was to synthesize sex-based differences in dating app use, app-mediated sexual behaviors, and sexual health outcomes using sex-stratified quantitative data from observational studies. This study was designed to address the following research question: Do women and men differ in the prevalence of dating app use and in app-mediated sexual behaviors and sexual health outcomes, including casual sexual encounters, sexual function, sexual satisfaction, sexual orientation, history of STIs, experience of sexual intercourse, and online versus offline partner-seeking behaviors?

## Methods

The aim of this systematic review and meta-analysis was to synthesize sex-based differences in dating app use, related sexual behaviors, and sexual health outcomes using sex-stratified quantitative data from observational studies. Dating applications have transformed contemporary sexual and relational practices by facilitating rapid partner selection, expanding sexual networks, and increasing opportunities for casual and non-committed sexual encounters. However, accumulating evidence indicates that these digitally mediated experiences are heterogeneous and may differ between women and men, reflecting broader social, behavioral, and contextual influences.

In this review, the terms sex and gender are conceptually distinguished but analytically aligned with the terminology used in the primary studies. All quantitative syntheses are based on sex-stratified data as reported by the original studies, which predominantly classified participants as women and men according to self-reported biological sex. Although some studies use the term gender, the extracted data reflect binary sex-based classifications rather than socially constructed gender identities or roles. Accordingly, the present analyses should be interpreted as examining sex-based differences in dating app–related behaviors and outcomes. This limitation of the existing evidence base is acknowledged and addressed in the Discussion.

Over the past decade, observational studies conducted across diverse cultural and social contexts have reported sex-stratified findings on dating app use, engagement in casual sexual encounters, partner-seeking behaviors, and STIs. Despite the growing volume of research, these findings remain fragmented and highly heterogeneous, and sex-based differences have not been systematically or quantitatively synthesized across outcome domains.

To address this gap, the present review focuses on adolescent and adult populations and adopts a comparative, sex-stratified meta-analytic approach to describe patterns of variation between women and men in dating app–related behaviors and outcomes. Rather than estimating causal effects, the analysis aims to summarize prevalence differences and assess the consistency of sex-based patterns across studies.

The study was conducted in accordance with the PRISMA 2020 reporting guidelines for systematic reviews and meta-analyses, and the protocol was prospectively registered in the PROSPERO international database (Registration number: CRD420261478391).

### Search strategy

A comprehensive and structured search strategy was developed to identify empirical studies reporting sex-stratified quantitative data on dating app–related sexual behaviors and associated health outcomes. The search focused on studies examining dating app use and app-mediated sexual experiences, including casual and non-committed sexual encounters, partner-seeking behaviors, sexual orientation distribution, and STIs, with outcomes reported separately for women and men.

We systematically searched PubMed/MEDLINE, Web of Science, PsycINFO, Scopus, and the Cochrane Library from January 1, 2008 to December 31, 2025. The start date was selected to coincide with the widespread global adoption of smartphone-based dating applications in the late 2000s, which fundamentally altered patterns of partner selection and digitally mediated sexual interaction. Studies published prior to this period predominantly examined offline casual sexual encounters using heterogeneous terminology and conceptual frameworks that were not directly aligned with contemporary app-based dating practices. Restricting the search to post-2008 literature therefore enhanced conceptual coherence and methodological comparability across studies. Extending the search through 2025 ensured inclusion of the most recent evidence and reduced the likelihood that the synthesis would become outdated at the time of publication.

The search strategy was developed in accordance with PRISMA 2020 recommendations and was tailored to each database using a combination of controlled vocabulary terms (eg, MeSH) and free-text keywords. Boolean operators and proximity commands were applied to optimize sensitivity while maintaining specificity.

Core search terms encompassed combinations of the following concepts:

“dating apps,” “online dating,” “mobile dating,” “app-based dating,” “Tinder,” “Grindr,” “Bumble,” “casual sex,” “one-night stand,” “hookup,” “non-committed sexual relationships,” “sexual behavior,” “sexual intercourse,” “partner-seeking,” “online dating behavior,” “offline dating behavior,” “sexual orientation,” “sex differences,” “gender differences,” and “sexually transmitted infections.”

An example PubMed search strategy was as follows:

(“Online Dating”[Mesh].

OR “dating app*.”

OR “mobile dating.”

OR “app-based dating.”

OR Tinder

OR Bumble

OR Grindr)

AND

( “Casual Sex.”

OR “one-night stand*.”

OR hookup*.

OR “non-committed sexual relationship*”.

OR “casual sexual encounter*”).

AND

( “Sexual Behavior”[Mesh].

OR “sexual intercourse.”

OR “partner seeking.”

OR “online dating behavior”.

OR “offline dating behavior”.

OR “Sexually Transmitted Diseases”[Mesh].

OR STI*

OR STD*).

AND

( “Sex Differences”[Mesh].

OR “gender difference*”

OR women

OR men)

Searches were restricted to quantitative, peer-reviewed studies published in English. Non-English publications were excluded unless sufficient methodological and outcome data were available in the English abstract to permit eligibility assessment. This language restriction may have introduced selection bias by underrepresenting studies from non-English-speaking regions, a limitation that is acknowledged in the interpretation of the findings, particularly given the culturally embedded nature of dating practices and sexual behavior.

To ensure comprehensive coverage, electronic database searches were supplemented by:

(1) manual screening of the reference lists of all included studies; and
(2) targeted hand-searching of key journals in the fields of sexuality research, relationship science, and digital health.

A systematic search of grey literature (eg, dissertations, conference proceedings, and industry reports) was not conducted, as such sources rarely provide extractable sex-stratified quantitative data suitable for meta-analysis and typically lack peer review. While this decision may have limited inclusion of emerging evidence, it enhanced the methodological robustness and comparability of the pooled estimates. Detailed database-specific search strategies and screening procedures are provided in Supplementary Material 1.

### Inclusion and exclusion criteria

#### Study eligibility and design

This systematic review and meta-analysis included quantitative observational studies (cross-sectional, cohort, and case–control designs) that reported sex-stratified data on dating app–related sexual behaviors and associated health outcomes. Eligible studies examined dating app use and/or dating app–mediated sexual experiences, with outcomes reported separately for women and men. Intervention studies and experimental designs were not considered, as the focus of the review was on describing sex-based differences rather than estimating causal effects.

**Population (P):** Studies involving adolescent and adult participants were eligible for inclusion. Adolescent samples were included where participants were aged 16 years or older and participation was legally and ethically permitted; adult samples included participants aged 18 years and older. No restrictions were applied with respect to country, cultural setting, or sexual orientation.

Studies that included mixed relationship or partner-seeking contexts were retained if data specific to dating app use or dating app–mediated sexual behaviors could be extracted separately by sex. Studies were excluded if sex-disaggregated data could not be derived.

**Exposure/context (I/E):** Eligible studies examined at least one of the following dating app–related contexts:

1. **Dating app use**, defined as engagement with mobile or web-based platforms designed to facilitate romantic or sexual connections (eg, Tinder, Bumble, Grindr, Hinge), regardless of whether sexual activity subsequently occurred; and/or
2. **Dating app–mediated casual sexual encounters**, defined as non-committed sexual interactions (eg, one-night stands or hookups) that were explicitly initiated through dating applications.

Given that dating app use and casual sexual engagement often overlap in real-world settings, studies addressing both contexts were included. For analytic purposes, studies were classified according to the primary context assessed (dating app use versus app-mediated casual sexual encounters), and this distinction was considered descriptively and, where relevant, in subgroup or meta-regression analyses.

**Comparison (C):** A formal control group was not required for inclusion. Studies were eligible if they provided extractable sex-stratified quantitative data allowing comparison between women and men. Where available, comparisons included differences between women and men among dating app users or among individuals engaging in app-mediated casual sexual encounters.

Studies without explicit comparison groups were retained if sufficient sex-disaggregated data were reported to allow calculation of prevalence estimates or comparative effect measures between women and men.

**Outcomes (O):** Studies were included if they reported at least one of the following outcomes stratified by sex:

- prevalence of dating app use;
- engagement in dating app–mediated casual sexual encounters;
- sexual satisfaction or sexual function outcomes among dating app users;
- experience of sexual intercourse;
- distribution of sexual orientation among dating app users;
- prevalence or history of STIs reported in relation to dating app use.

Given the descriptive and comparative focus of the review, outcomes were synthesized using prevalence proportions or sex-based comparative effect estimates as reported in the original studies, without attempting to harmonize outcome definitions across studies.

**Study design (S):** Quantitative observational studies were eligible for inclusion. Mixed-methods studies were included only if quantitative results were reported separately and provided extractable sex-stratified data. The following were excluded: Intervention studies, qualitative-only studies, case reports, conference abstracts without full data, narrative reviews, editorials, commentaries, and theoretical papers.

**Exclusion criteria:** Studies were excluded if they met any of the following criteria:

- did not distinguish dating app–mediated sexual behaviors from offline casual sexual encounters and did not provide extractable app-specific data;
- focused exclusively on romantic relationship formation, partner matching, or relationship satisfaction without assessment of sexual behaviors or sexual health outcomes related to dating app use;
- examined commercial sex work or transactional sexual activities not facilitated through dating applications;
- relied solely on qualitative methods or mixed-methods designs without separable quantitative data;
- lacked sufficient methodological detail to permit reliable assessment of eligibility, data extraction, or risk of bias.

### Study selection and data extraction

All records identified through the electronic database searches were imported into EndNote 21 (Clarivate, Philadelphia, PA, USA) for reference management and duplicate removal prior to screening. Study selection was conducted independently by 2 reviewers in line with PRISMA 2020 guidelines using a multi-stage screening process. In the first stage, titles and abstracts were screened to remove clearly irrelevant records. Full texts of potentially eligible studies were then retrieved and assessed in detail against the predefined inclusion and exclusion criteria. To ensure completeness, reference lists of all included studies and relevant review articles were also screened manually to identify additional eligible studies that may not have been captured through database searches. Any disagreements between reviewers were resolved through discussion, and when necessary, by consultation with a senior researcher until consensus was reached.

Data extraction was performed independently by 2 reviewers using a standardized and piloted data extraction form. This approach was used to ensure consistency and transparency across studies. Extracted information included basic study characteristics (authors, year of publication, and country), study design, recruitment and sampling methods, study setting, sample size, participant age characteristics, and sex distribution.

Additional information specific to dating app use was extracted where available. This included the type of dating application or platform examined (eg, Tinder, Bumble, Grindr), indicators of dating app use such as current or lifetime use and frequency of use, and details of app-mediated sexual behaviors, including casual sexual encounters, one-night stands, and non-committed sexual relationships.

Quantitative outcome data were extracted for all eligible sex-stratified outcome domains examined in the review. These included the prevalence of dating app use, engagement in dating app–mediated casual sexual encounters, sexual satisfaction or sexual function outcomes, experience of sexual intercourse, online versus offline partner-seeking behaviors, distribution of sexual orientation among dating app users, and prevalence or history of STIs. For each outcome, the manner in which results were reported (eg, prevalence estimates, raw counts, or comparative statistics) was documented. Studies reporting outcomes separately for women and men were extracted directly, whereas studies that did not provide sex-disaggregated data were excluded from quantitative synthesis.

Where effect estimates were reported, these were extracted as presented in the original studies, including prevalence proportions, odds ratios (ORs), or risk ratios with corresponding confidence intervals (CIs). All extracted data were cross-checked by a second reviewer to ensure accuracy and internal consistency. When essential information was missing or unclear, attempts were made to contact the original study authors for clarification. Studies for which the required sex-stratified data could not be obtained were retained for narrative description where appropriate but were excluded from quantitative pooling.

### Risk of bias and methodological quality assessment

The methodological quality and risk of bias of included studies were assessed using the Joanna Briggs Institute (JBI) Critical Appraisal Checklist for Analytical Cross-Sectional Studies. This tool was selected because the majority of included studies employed observational, predominantly cross-sectional designs and reported sex-stratified prevalence or comparative data.

The JBI checklist evaluates key sources of potential bias in cross-sectional research, including the appropriateness of the sampling frame, adequacy of participant recruitment, validity and reliability of exposure and outcome measurement, identification and management of confounding factors, and appropriateness of statistical analyses. Each item was rated as “yes,” “no,” “unclear,” or “not applicable.” In keeping with JBI guidance, checklist items were not combined into a numerical quality score; instead, overall judgements were based on patterns across domains.

To support transparent reporting, risk-of-bias judgements were summarized visually using Review Manager (RevMan) software. Although RevMan’s standard tools are designed for randomized controlled trials, the software was used in this review solely to present JBI-derived assessments in traffic-light plots and summary figures. RevMan was not used as the appraisal instrument itself.

Study quality was not used as a criterion for exclusion. Rather, risk-of-bias assessments were considered when interpreting the findings and assessing the robustness of pooled estimates. Particular attention was paid to participant selection and control of confounding, which represent common methodological challenges in observational research on dating app–related sexual behaviors.

### Statistical analysis

All statistical analyses were conducted using random-effects meta-analytic models, acknowledging the substantial methodological and contextual heterogeneity inherent in observational studies examining dating app–related behaviors. Given that the included studies were predominantly cross-sectional and did not involve experimental manipulation of exposure, random-effects models were considered the most appropriate and conservative approach.

For all outcomes, sex-based comparative analyses were performed, with women and men serving as the comparison groups. Binary outcomes were synthesized using ORs with corresponding 95% CIs. For each study, sex-stratified event counts and total sample sizes were extracted, and pooled ORs were calculated using the Mantel–Haenszel method under a random-effects assumption. An OR below 1.0 indicated a lower prevalence of the outcome among women compared with men, whereas an OR above 1.0 indicated a higher prevalence among women.

Separate meta-analyses were conducted for each outcome domain, including dating app use, engagement in dating app–mediated casual sexual encounters, experience of sexual intercourse, online versus offline partner-seeking behaviors, distribution of sexual orientation among dating app users, and prevalence of STIs. Outcomes were analyzed independently to avoid inappropriate pooling across conceptually distinct constructs.

Between-study heterogeneity was assessed using the *I*^2^ statistic, Cochran’s Q test, and the between-study variance (τ^2^). Given the consistently high levels of heterogeneity observed across analyses, pooled estimates were interpreted as average effects across heterogeneous study populations rather than precise effect sizes applicable to all contexts.

Sensitivity analyses were performed using a leave-one-out approach by sequentially excluding individual studies to assess the robustness of the pooled estimates and to determine whether any single study disproportionately influenced the overall results or the observed heterogeneity. Studies were not excluded based on methodological quality alone; however, findings were interpreted in light of the overall risk-of-bias assessments.

Assessment of publication bias was limited by the observational nature of the evidence base and the relatively small number of studies contributing to several outcomes. For outcome domains with a sufficient number of studies, funnel plots were visually inspected for asymmetry. Formal statistical tests for publication bias were applied selectively and interpreted with caution.

All statistical analyses were conducted using RevMan version 5.4.1 (The Cochrane Collaboration, London, UK), and results were presented as forest plots displaying individual study estimates, study weights, and pooled effect sizes under random-effects models.

### Publication bias

Publication bias was examined using both visual and statistical approaches. Funnel plots were inspected for asymmetry for all outcomes with a sufficient number of studies, recognizing that asymmetry may also arise from between-study heterogeneity or small-study effects. When at least 10 studies contributed to a pooled estimate, formal tests for funnel-plot asymmetry were conducted to evaluate the presence of small-study bias. Where applicable, sensitivity analyses were performed to assess the stability of pooled results after excluding studies contributing to potential asymmetry. Publication bias assessments were interpreted cautiously given the observational nature of the evidence base and variability in study precision.

### Ethics statement

Ethical approval was not required for this study because it is a systematic review and meta-analysis based exclusively on data extracted from previously published studies. No new human participants were recruited, and informed consent was therefore not applicable.

## Results

### Study selection

**Figure 1:**
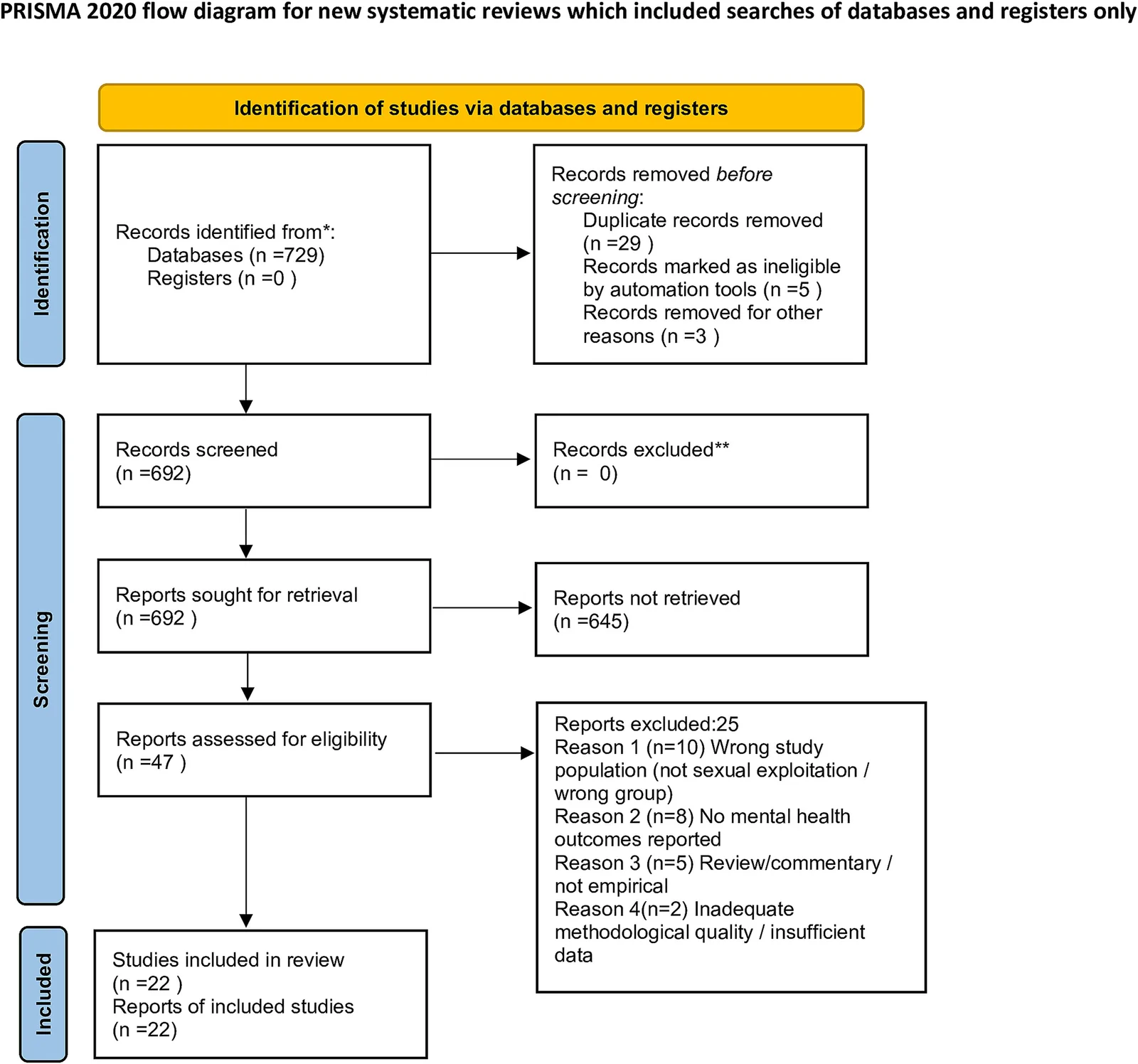
PRISMA flow diagram. PRISMA: Preferred Reporting Items for Systematic Reviews and Meta-Analyses.

Figure 1 presents the PRISMA 2020 flow diagram summarizing the literature search and study selection process. A total of 729 records were identified through systematic database searches. After removal of duplicate records (*n* = 29), automated exclusions (*n* = 5), and other clearly irrelevant records (*n* = 3), 692 records remained for title and abstract screening.

 Following this initial screening, 645 records were excluded because they did not meet the predefined inclusion criteria, primarily due to lack of relevance to dating application use, absence of sexual or psychological outcome measures, or non-quantitative study designs.

Full-text eligibility assessment was conducted for 47 articles. Of these, 25 studies were excluded for reasons including wrong population, inability to distinguish dating app–mediated behaviors from offline sexual behaviors, absence of relevant sexual or psychological outcomes, or non-empirical study design.

Ultimately, 22 studies met all inclusion criteria and were included in the final systematic review and meta-analysis.

### Included studies

The initial database search yielded a substantial number of records across all electronic databases and was supplemented by citation tracking and manual screening of reference lists. Following the removal of duplicate records, titles and abstracts were screened against predefined eligibility criteria. Potentially relevant records underwent full-text assessment. After detailed evaluation, 22 studies met all inclusion criteria and were retained for the final qualitative and quantitative synthesis (Figure 1).

The included studies demonstrate broad geographical and cultural diversity, with research conducted across North America, Europe, Australia, South Asia, East Asia, and Latin America. Studies from North America and Europe primarily examined dating app use among university students and community samples, focusing on sexual behaviors, condom use, and psychosocial correlates.^7–10^ Research from Australia frequently drew on event-based or population-level samples, including individuals recruited at music festivals or through sexual health surveillance systems.^11–13^ Studies from Latin America and South Asia provided important contextual insights into dating app use and sexual risk behaviors in emerging and non-Western sociocultural settings.^14–17^

Most included studies employed observational designs, predominantly using cross-sectional survey methodologies. Samples were recruited from diverse settings, including universities and colleges,^18^^,^^19^ community-based or online platforms,^20^^,^^21^ music festival or nightlife environments,^12^^,^^13^ and clinical contexts, such as sexual health or dermatology clinics.^7^^,^^17^ Several studies focused on populations considered to be at elevated sexual health risk, including adolescents and young adults, patients attending STI clinics, and individuals reporting frequent or problematic dating app use.^22–24^

Across studies, the primary exposure was operationalized as dating application use, including current or lifetime use, frequency or intensity of engagement, platform-specific use (eg, Tinder or Bumble), or motivations for dating app use.^15^^,^^19^^,^^20^ In several studies, exposure also encompassed dating app–mediated casual sexual encounters, such as hookups, one-night stands, or non-committed sexual relationships explicitly facilitated through dating applications.^21^^,^^25^^,^^26^ Although operational definitions varied, all included studies reported extractable quantitative data stratified by sex, allowing synthesis of sex-based differences.

Reported outcomes clustered into several interrelated domains. Sexual behavior and sexual health outcomes included engagement in casual sex, number of sexual partners, condom use practices, contraceptive awareness or use, and prevalence or history of STIs.^7^^,^^21^^,^^25^^,^^27^ Dating-related behavioral outcomes captured patterns of online versus offline partner-seeking, methods of meeting sexual partners, and characteristics of dating app engagement.^8^^,^^11^^,^^12^ A subset of studies additionally examined relational and psychosocial outcomes, including attachment-related experiences, sexual satisfaction, match-related need satisfaction, problematic dating app use, and indicators of psychological well-being or distress.^9^^,^^23^^,^^28^ Collectively, this literature provides a multidimensional overview of how digitally mediated dating contexts intersect with sexual behavior, relational processes, and health-related outcomes.

Studies excluded at the full-text stage were removed for reasons consistent with PRISMA 2020 standards. The most common reasons for exclusion included lack of extractable sex stratified quantitative data, failure to distinguish dating app mediated behaviors from offline sexual activity, absence of relevant sexual or behavioral outcomes, qualitative-only or theoretical designs, and insufficient methodological detail to permit reliable quantitative synthesis. This rigorous selection process ensured that only studies with appropriate quantitative data were included, thereby strengthening the interpretability and robustness of the pooled estimates.

### Study and participant characteristics

The final synthesis included 22 quantitative studies published between 2016 and 2025, examining dating app use and dating app mediated sexual behaviors across diverse populations and settings (Table 1). The majority of studies were conducted within the past decade, reflecting the rapid expansion and normalization of mobile dating platforms in contemporary relationship formation.^7^^,^^19^^,^^21^ Sample sizes varied substantially, ranging from fewer than 200 participants in clinic-based or subgroup-focused studies to several thousand participants in large university or community surveys.^11^^,^^12^^,^^20^ Across all studies, participants were predominantly adolescents and young adults, although several reports included broader adult age ranges.^9^^,^^10^^,^^18^

**Table 1:** **Characteristics of the participants of the included studies (*n* = 22)**.

**Author, year, country**	**Study desing**	**Sample size** ***Total (N), Female\Male (n)***	**Age**	**Popoulation type**	**Sexual minority focus**	**Dating App(s)**	**Definition of dating app use**	**Outcome(s) reported**
Andersson et al. 2019, Sweden	Cross-sectional, clinic-based survey	*N* = 941 female = 367 male = 574	Mean: 29 Range: 18-67	Patients visiting the STI clinic	No	Dating apps (eg, Tinder)	Dating app use in the past 12 months to find a temporary sexual partner	Dating app use; *Chlamydia trachomatis* prevalence; number of sexual partners; risky sexual behaviors
Blanc, 2024, Spain	Cross-sectional, online survey	*N* = 2288 female = 1380 male = 908	Mean: 23.23 SD: 3.87 Range: 18-35	University students	Yes (lesbian, gay, and bisexual (LGB) individuals, heterosexual)	Tinder, Badoo, Meetic, Lovoo, Grindr, other	Ever use of dating apps (yes/no); frequency and number of uses	Dating app use; sociodemographic characteristics; sexual attitudes; sexual risk behaviors; number of sexual partners; condom use
Castro et al., 2020, Spain	Cross-sectional, online survey	*N* = 1705 female = 70% (~1194) male = 30% (~511)	Mean: 20.60 SD: 2.09 Range: 18-26	University students	Yes (sexual minority, heterosexual)	Dating apps (Tinder, Grindr or similar)	Current (last 3 months), previous, or non-use of dating apps	Sociodemographic characteristics; personality traits (Big Five, Dark Core); dating app use.
Choi et al., 2016, China	Cross-sectional, campus-based survey	*N* = 666 female = NR male = NR	Mean: 20.03 SD: 1.52	University students	Yes (homo/bisexual, heterosexual)	Dating apps (GPS-based smartphone apps)	Dating app use and duration (no use; <1 month, 1-2 months, 3-12 months, >12 months)	Dating app use; condom use; casual sex encounters; sexual risk behaviors.
Coffey et al., 2022, USA	Cross-sectional, online survey	*N* = 247 female = 40.49% (~100) male = 59.51% (~147)	Mean: 27.34 SD: 4.47	Single adults	No	Tinder or other online dating sites	Dating app use (yes/no) and frequency	Attachment styles; permissiveness; searching for partners; feelings following sexual experiences
Constantinou et al., 2021, Australia	Cross-sectional, clinic-based survey	*N* = 698 female = 373 male = 325	Median: 28 (IQR 24-35) for men and 25 (IQR 23-29) for women	Clients aged 16 years or older	No	Dating apps (eg, Tinder)	Dating app use (yes/no)	Methods of meeting sexual partners; number and type of sexual partners; sexual practices and drug use; STI positivity
Flesia et al., 2021, Italy	Cross-sectional, online survey	*N* = 1278 female = 814 male = 464	Mean: 27.94 SD: 7.85	Italian respondents	Yes (homosexual, heterosexual, other)	Online smartphone dating applications based on geosocial networking	Dating app use (active, former, non-use), duration, motives, and frequency.	Sociodemographic characteristics; dating app use; number of protected and unprotected sexual partners; frequency of hook-ups; lifetime STI history
Garga et al., 2021a, Australia (JMIR)	Cross-sectional, music festival-based survey	*N* = 862 female = 558 male = 302	Mean: NR Age 18-20 *n*:383 (44.4%), Age 21-24 *n*:395 (45.8%), Age 25-30 *n*:558 (64.9%)	The music festival attendees	Yes (heterosexual, nonheterosexual)	Geosocial networking dating apps	Dating app use in the last 12 months (yes/no)	Geosocial networking dating apps use; contraception; specific risky sexual behaviors; and related risk perceptions with dating app use
Garga et al., 2021b, Australia (HRJ)	Cross-sectional, paper-based survey	*N* = 448 female = 291 male = 156	Mean: NR Age 18-20 *n*:192 (42.9%), Age 21-24 *n*:215 (48.0%), Age 25-30 *n*:41 (9.2%)	Festival patrons aged 18-30	Yes (heterosexual, homosexual, bisexual, other)	Tinder, Coffee meets bagel, Grindr, Bumble, OkCupid, Hinge, Plenty of fish, Happn, RSVP, eHarmony, Zoosk, other	Dating app use (yes/no) and dating app use at festivals (yes/no)	Reasons for using dating apps; dating apps use; duration of dating apps use; dating app relationships; unintended consequences; change in sexual behaviors; condom use; STI status
Huang et al., 2023, Taiwan	Cross-sectional, paper-based survey	*N* = 2595 female = 1369 male = 1226	NR	Adolescents (Seventh-grade students from middle schools)	No	Dating apps (unspecified)	Dating app use and frequency (never, ever before a year, a few times within a year, a few times within a month, a few times within a week)	Dating app use; online victimization (cyberbullying, sexual harassment); psychological distress; marketing exposure; parental mediation; adolescent characteristics
Joseph et al., 2025, India	Cross-sectional, community-based	*N* = 270 female = 110 male = 160	Mean: NR Age 18-24 *n*:99 (36.6%), Age 25-34 *n*:163 (60.3%), Age 35-44 *n*:8 (2.96%)	Adults using online dating apps	No	Tinder, Bumble, Happn, Hinge, Okcupid, Others (Instagram, Snapchat, Facebook, etc.)	Current dating app use and frequency (daily, weekly, monthly, rarely)	Sociodemographic characteristics; contraceptive use and awareness; dating app use
Liberacka-Dwojak et al., 2023, Poland	Cross-sectional psychometric validation study	Study 1; *N* = 271 female = 186 male = 85 Study 2; *N* = 162 female = 115 male = 47	Study 1; Mean: 25.06 SD: 5.37 Range: 18-52 Study 2; Mean: 25.07 SD: 5.37 Range: 18-52	Adults using Tinder	Yes (heterosexual, homosexual, bisexual, other)	Tinder	Current Tinder use (app version and average weekly use time)	Problematic Tinder use; safe sex behaviors; sociodemographic characteristics
Martinez et al., 2025, Brazil	Cross-sectional, web-based survey	*N* = 487 female = 68.9% (~336) male = 31.1% (~151)	Mean: 22.2 SD: 3.1 Range: 18-46	Undergraduate students	Yes (heterosexual, homosexual, bisexual)	Tinder, Grindr, Hornet, Bumble, Fem Dating, Happn, Instagram, OkCupid, Scruff, Umatch, and Zoe	Dating app use (yes/no)	Sociodemographic characteristics; dating app use; sexual risk behaviors; smoking, alcohol, and drug use.
Menon, 2024, India	Cross-sectional, online survey	*N* = 298 female = 109 male = 189	Mean: 22.23; SD: 2.71 Range = 18-29	Emerging adults	No	Bumble	Bumble use during the past six months.	Bumble use; motivations for Bumble use; sociodemographic characteristics
Reeves et al, 2024, USA	Cross-sectional, online survey	*N* = 122 female = 53 male = 66 other = 3	Mean: 23.3 SD: 3.5	Young adult college students	Yes (Heterosexual, gay/lesbian, bisexual and other)	Dating apps (unspecified)	Ever use of dating apps (yes/no)	Sociodemographic characteristics; sexual and risky sexual behaviors; dating app use.
Sumter, 2019, Netherlands	Cross-sectional	*N* = 541 female = 60.1% (~325) male = 39.9% (~216)	Mean: 23.71 SD: 3.29 Range: 18-30	Emerging adults	Yes (heterosexual, non-heterosexual)	Dating apps (eg, Tinder, Grindr, Happn, and Scruff)	Dating app use (a couple of times a month or more)	Dating app use; dating app motivations; dating anxiety; sensation seeking; sexual permissiveness
Tavares et al., 2020, Brazil	Cross-sectional study	*N* = 359 female = 173 male = 186	Median: 21	College Students	Yes (heterosexual, homosexual, bisexual)	Dating apps (unspecified)	Ever use of dating apps (yes/no) and casual encounters with partners met via the app	Sociodemographic characteristics; dating app use; sexual behaviors and attitudes.
Thind et al., 2025, India	Cross-sectional, paper‑based survey	*N* = 150 female = 39 male = 111	Mean: 30.57 SD: 8.9 Range: 17- 63	Patients of STIs	Yes (attracted to the opposite sex, same‑sex, or both sexes)	Tinder Grindr Bumble Hinge Facebook Instagram	Dating app use (yes/no), purpose, number of apps used, duration of use	Dating app usage; sexual behaviors; and STI diagnoses
Tomaszewska & Schuster, 2020, Germany	Cross-sectional, online survey	*N* = 491 female = 295 male = 196	Mean: 25.9 SD: 4.22	Dating app users and non-users	No	Dating apps (eg, Tinder)	Ever use of dating apps (yes/no); frequency	Dating app use; risky sexual scripts; risky sexual behaviors; sexual self-esteem; sexual assertiveness; acceptance of sexual coercion
Wang et al., 2023, China	Cross-sectional, online survey	*N* = 582 female = 271 male = 311	Mean: 25.39 SD: 3.66	Chinese heterosexual dating app users	No	Dating apps (eg, Tantan, Momo)	Currently using dating apps	Casual sex motivation for dating app use; number of sexual partners met via dating apps; risky sexual behaviors; STI diagnoses.
Vranken et al., 2024, Belgium	Multi-method study (content analysis & cross-sectional studies)	Study 2; *N* = 325 female = 80.45% (~261) male = 19.55% (~64) Study 3; *N* = 323 female = 73.68% (~238) male = 26.32% (~85)	Study 2; Mean: 22.45 SD: 2.14 Study 3; Mean: 23.31 SD: 2.79	Emerging adults	Yes (exclusively heterosexual, not exclusively heterosexual)	Dating apps (ie, Tinder)	Currently using (a) dating app(s)	Dating app use frequency; swiping frequency; mate value preferences sexual satisfaction, need satisfaction
Naranjo-Márquez, 2025, Spain	Cross-sectional, online survey	*N* = 730 female = 511 male = 209 other = 10	Median: 21 Range: 20-22	University students of the health sciences	Yes (gay or lesbian, heterosexual, bisexual)	Dating apps (unspecified)	Use of dating apps (yes/no)	Use of dating apps; drug and pornographic material consumption; and STI history

NR: Not reported

Most studies recruited participants from educational institutions, community samples, or online platforms, particularly those examining dating app use among university students or emerging adults.^8^^,^^14^^,^^16^^,^^22^ A smaller subset of studies drew samples from sexual health or dermatology clinics, especially in investigations focusing on STIs and sexual risk behaviors.^7^^,^^17^ Several studies explicitly targeted or oversampled sexual minority populations, including men who have sex with men and individuals identifying as lesbian, gay, bisexual, or other sexual orientations, whereas others examined predominantly heterosexual samples.^21^^,^^23^^,^^24^

Dating app exposure was assessed using a range of indicators, including current or lifetime app use, frequency or intensity of use, and platform-specific engagement (eg, Tinder, Bumble, Grindr).^15^^,^^19^^,^^20^ In studies focusing on sexual behavior, casual sexual encounters were commonly operationalized as one-night stands, hookups, or non-committed sexual relationships explicitly facilitated through dating applications.^12^^,^^25^^,^^26^ Although measurement approaches differed across studies, all included reports provided sex-disaggregated quantitative data, allowing comparisons between women and men.

Reported outcomes varied across studies but consistently reflected dating-related sexual behaviors and health indicators. Sexual health outcomes included STI diagnoses, condom use, contraceptive awareness or practices, and patterns of sexual risk behavior.^7^^,^^21^^,^^27^ Behavioral outcomes captured differences in partner-seeking strategies, online versus offline meeting contexts, and sexual activity patterns.^8^^,^^10^^,^^11^ In addition, several studies reported relational and psychosocial correlates of dating app use, such as relationship intentions, attachment-related experiences, sexual satisfaction, need satisfaction with matches, problematic dating app use, and selected indicators of psychological well-being or distress.^9^^,^^23^^,^^28^

Taken together, the included studies represent a heterogeneous yet coherent body of evidence spanning multiple regions, populations, and dating app contexts. This diversity provided the empirical foundation for the sex-based meta-analyses and heterogeneity assessments reported in the Results section, while also supporting exploration of contextual variation across studies.

### Primary outcome

#### Dating app use by sex

Twenty-two studies contributed data to the meta-analysis examining sex-based differences in dating app use. Across these studies, a total of 6660 women and 5909 men were included, of whom 2318 women and 2893 men reported using dating applications.

The pooled random-effects analysis indicated that women were less likely to report dating app use compared with men (OR = 0.46, 95% CI, 0.28-0.77). This finding suggests a lower reported prevalence of dating app use among women relative to men across the included studies.

**Figure 2:**
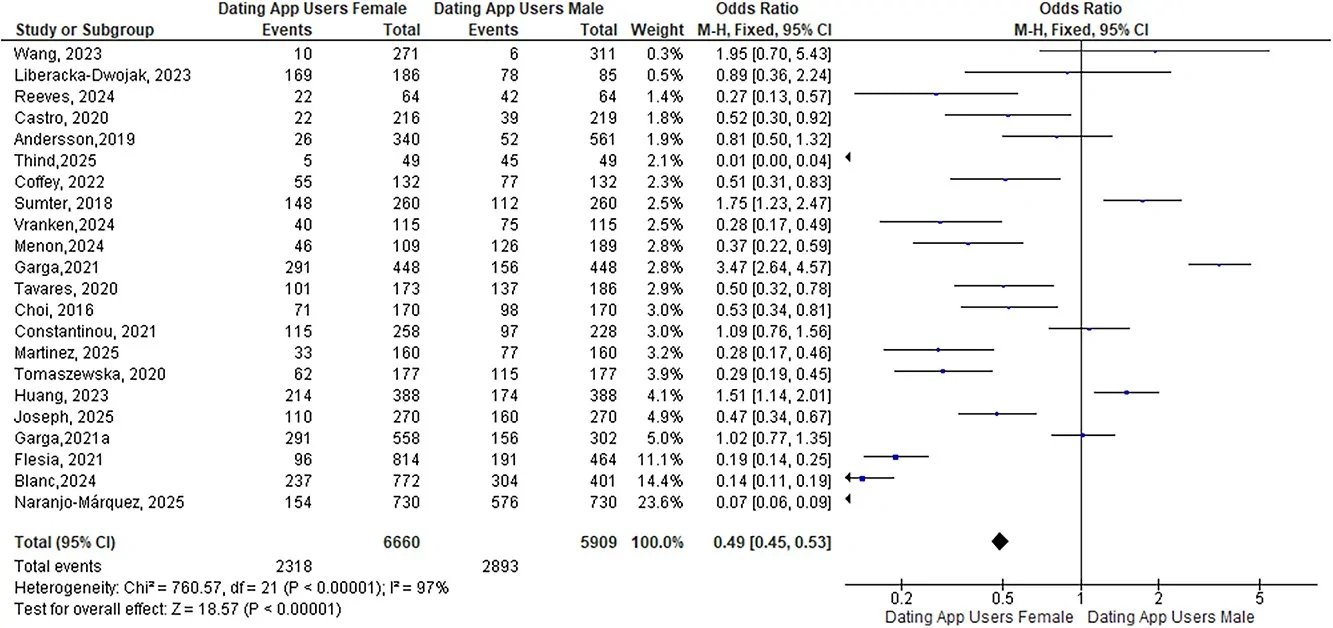
Forest plot comparing dating app use between males and females.

However, substantial between-study heterogeneity was observed (τ^2^ = 1.39; χ^2^ = 760.57, df = 21, *P* < .00001; *I*^2^ = 97%), indicating marked variability in the magnitude and direction of sex-based differences across individual studies (Figure 2).

#### Sexual function by sex

Six studies contributed data to the meta-analysis examining sex-based differences in sexual function among dating app users, comprising 1663 women and 1353 men. Across these studies, 469 women and 728 men met the study-specific criteria for sexual function impairment or dysfunction.

Using a random-effects Mantel–Haenszel model, the pooled analysis demonstrated a statistically significant sex difference in sexual function outcomes. Women who used dating applications were significantly less likely to report sexual function impairment compared with men (OR = 0.37, 95% CI, 0.20-0.66; Z = 3.33, *P* = .0009), corresponding to approximately 63% lower odds of impaired sexual function among women.

Between-study heterogeneity was substantial (τ^2^ = 0.43; χ^2^ = 41.15, df = 5, *P* < .00001; *I*^2^ = 88%), indicating considerable variability in effect estimates across studies. This heterogeneity likely reflects differences in study populations, cultural contexts, and operational definitions of sexual function, as well as the use of different measurement instruments and thresholds. Consequently, pooled estimates were interpreted as average effects across heterogeneous samples rather than as uniform sex differences applicable to all settings.

**Figure 3:**
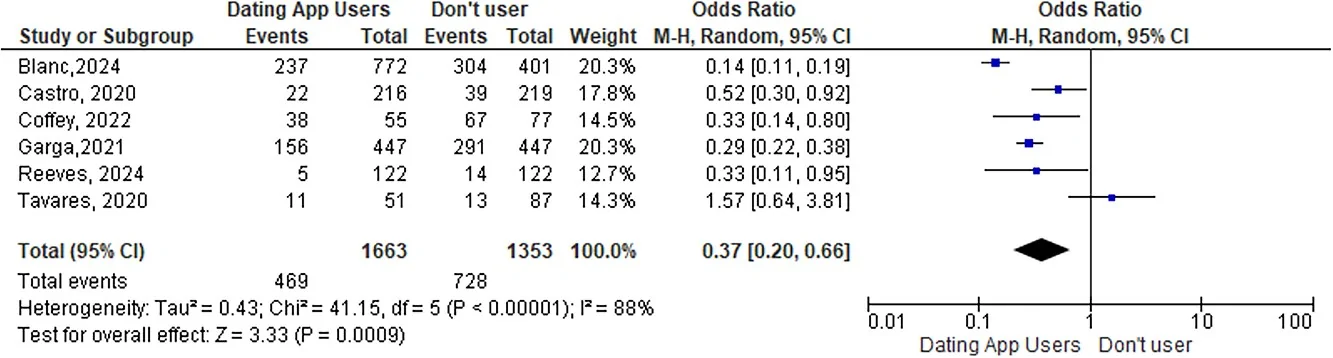
Sex-based differences in sexual function among dating app users.

Despite the high heterogeneity, the direction of effect was broadly consistent, with most studies reporting higher odds of sexual function impairment among men compared with women (Figure 3).

#### Casual sexual encounters by sex

Seven studies contributed data to the meta-analysis examining sex-based differences in engagement in dating app–mediated casual sexual encounters, comprising 1348 women and 1422 men. Across these studies, 660 women and 548 men reported engagement in casual or non-committed sexual encounters facilitated through dating applications.

Using a random-effects Mantel–Haenszel model, the pooled analysis indicated no statistically significant difference between women and men in the likelihood of engaging in casual sexual encounters (OR = 2.11, 95% CI, 0.96-4.63; Z = 1.86, *P* = .06). Although the point estimate suggested higher odds among women, the CI was wide and crossed the null, indicating substantial uncertainty around the pooled effect.

Between-study heterogeneity was very high (τ^2^ = 0.93; χ^2^ = 81.74, df = 6, *P* < .00001; *I*^2^ = 93%), reflecting pronounced variability in effect estimates across studies. This heterogeneity likely arises from differences in how casual sexual encounters were defined and measured (eg, one-night stands, hookups, multiple partners), as well as variation in study populations, cultural contexts, and recruitment settings.

Visual inspection of the forest plot revealed marked dispersion in study-specific ORs, including several studies reporting extreme estimates with wide CIs, particularly among smaller samples. These outliers contributed disproportionately to between-study heterogeneity and reduced the precision of the pooled estimate.

**Figure 4:**
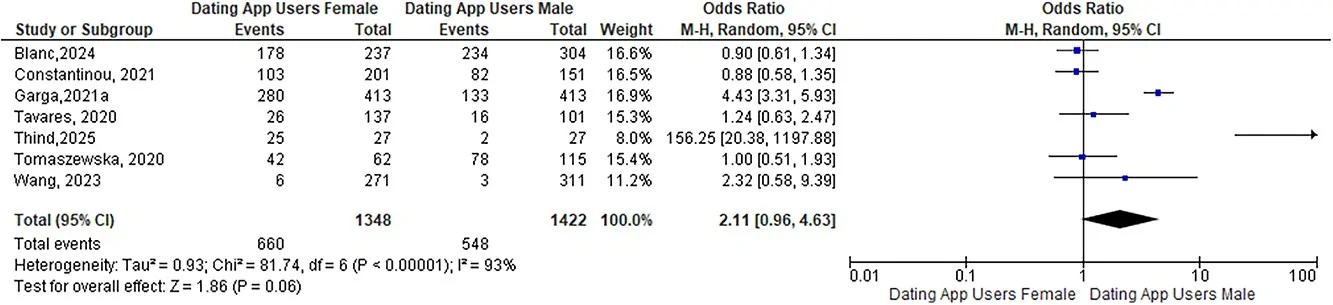
Sex-based differences in dating app‐mediated casual sexual encounters.

Overall, while individual studies reported sex differences in both directions, the pooled evidence does not support a consistent or statistically robust sex-based difference in dating app–mediated casual sexual encounters (Figure 4).

#### History of sexually transmitted infections by sex

Seven studies contributed data to the meta-analysis examining sex-based differences in self-reported history of STIs among dating app users, comprising 1467 women and 1878 men. Across these studies, 349 women and 549 men reported a previous diagnosis or history of an STI.

Using a random-effects Mantel–Haenszel model, the pooled analysis showed no statistically significant difference between women and men in the likelihood of reporting a history of STIs (OR = 0.52, 95% CI, 0.18-1.53; Z = 1.19, *P* = .23). Although the point estimate suggested lower odds of reported STI history among women, the CI was wide and crossed the null value, indicating substantial uncertainty around the pooled effect.

Between-study heterogeneity was very high (τ^2^ = 1.96; χ^2^ = 202.68, df = 6, *P* < .00001; *I*^2^ = 97%), reflecting pronounced variability across studies. This heterogeneity likely arises from differences in STI measurement methods (self-reported history versus clinic-based diagnosis), variation in recall periods, differences in sexual health service access, and heterogeneity in study populations and cultural contexts.

Inspection of the forest plot revealed marked dispersion in study-specific ORs, including both protective and risk estimates, with several studies contributing wide CIs. These findings suggest that contextual and methodological factors, rather than sex alone, play a major role in shaping reported STI history among dating app users.

**Figure 5:**
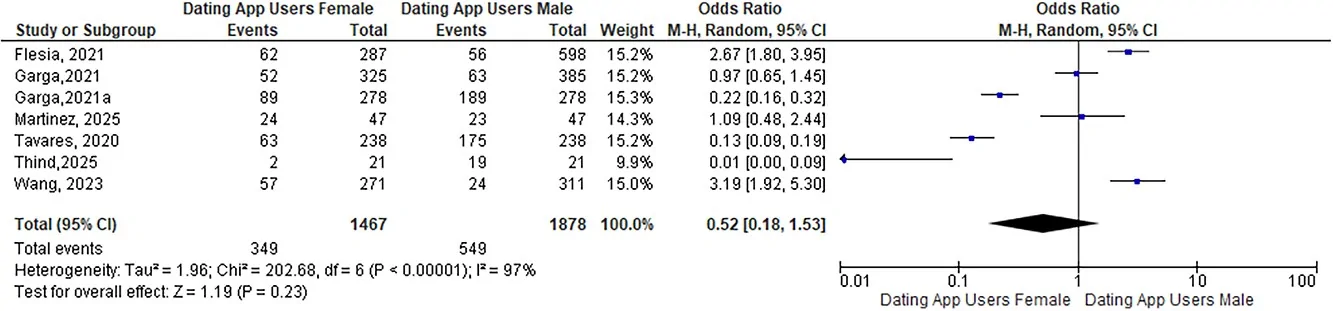
Sex-based differences in history of sexually transmitted infections among dating app users.

Overall, the pooled evidence does not support a consistent or statistically robust sex-based difference in history of STIs in the context of dating app use (Figure 5).

#### Sexual orientation by sex

Ten studies contributed data to the meta-analysis examining sex-based differences in reporting a sexual orientation (ie, non-heterosexual orientation) among dating app users, comprising 2331 women and 2247 men. Across these studies, 256 women and 491 men identified as having a sexual orientation other than heterosexual.

Using a random-effects Mantel–Haenszel model, the pooled analysis demonstrated a statistically significant sex difference in sexual orientation distribution among dating app users. Women were significantly less likely than men to report a sexual minority orientation (OR = 0.34, 95% CI, 0.16-0.69; Z = 2.96, *P* = .003), corresponding to approximately 66% lower odds of identifying as non-heterosexual among women compared with men in dating app–using populations.

Between-study heterogeneity was high (τ^2^ = 1.21; χ^2^ = 119.87, df = 9, *P* < .00001; *I*^2^ = 92%), indicating substantial variability across studies. This heterogeneity likely reflects differences in how sexual orientation was defined and measured (eg, identity-based versus behavior-based classifications), variation in cultural contexts and social acceptance of sexual minority identities, and differences in dating app platforms included in the studies (eg, general versus sexual minority–focused applications).

**Figure 6:**
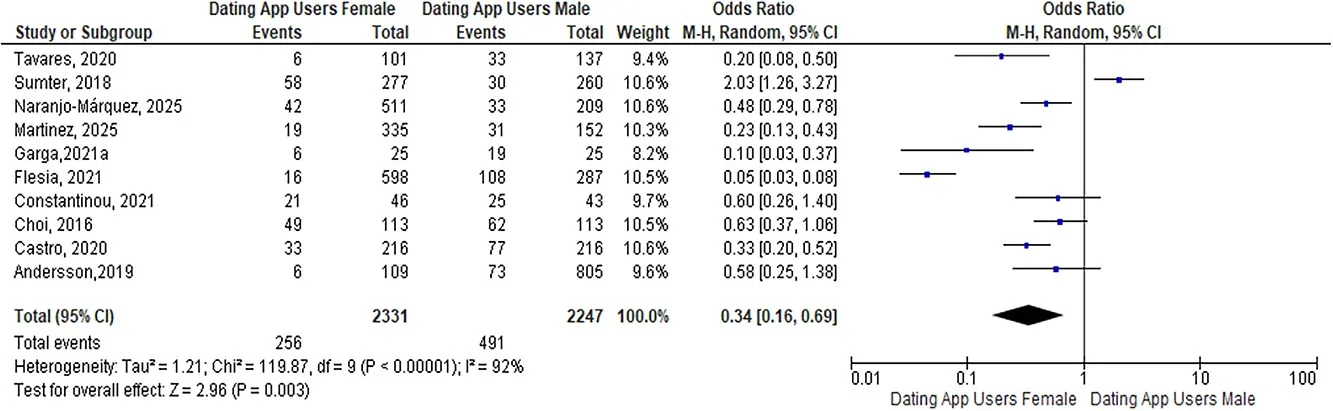
Sex-based differences in sexual minority orientation among dating app users.

Despite this heterogeneity, the direction of effect was consistent across most studies, with higher proportions of sexual minority identification observed among men than women (Figure 6).

#### Online vs offline partner-seeking among dating app users

Four studies contributed data to the meta-analysis comparing online versus offline partner-seeking behaviors among dating app users, comprising 1631 offline-oriented users and 1698 online-oriented users. Across these studies, 969 offline users and 963 online users reported the outcome of interest as defined in the original studies.

Using a random-effects Mantel–Haenszel model, the pooled analysis showed no statistically significant difference between online and offline dating app users (OR = 1.70, 95% CI, 0.18-15.83; Z = 0.47, *P* = .64). Although the point estimate suggested higher odds among offline-oriented users, the CI was extremely wide and crossed the null value by a large margin, indicating substantial imprecision in the pooled estimate.

Between-study heterogeneity was extreme (τ^2^ = 5.15; χ^2^ = 549.83, df = 3, *P* < .00001; *I*^2^ = 99%), reflecting pronounced variability across studies. This heterogeneity likely arises from differences in how “online” and “offline” partner-seeking were operationalized, variation in study populations and contexts, and the small number of contributing studies. Several studies reported highly divergent effect sizes, which contributed disproportionately to the wide CIs and limited interpretability of the pooled result.

**Figure 7:**
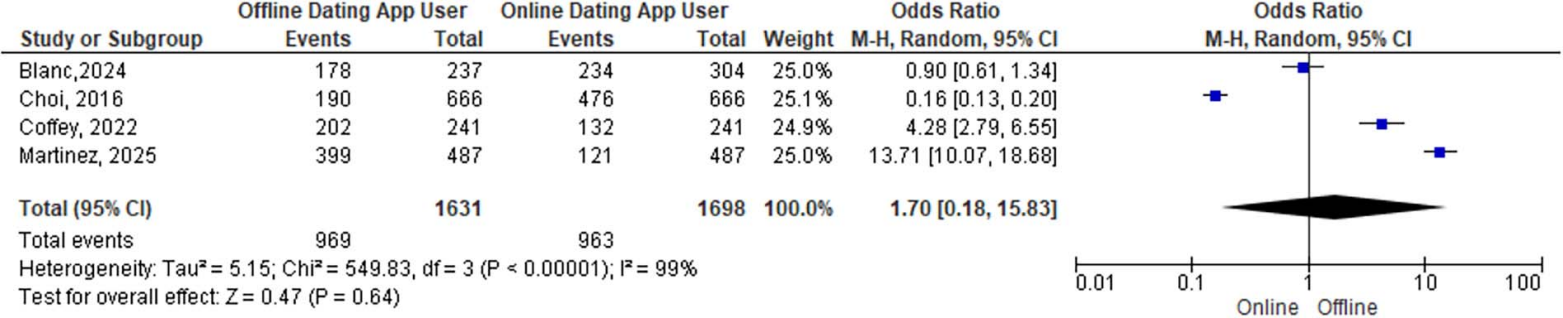
Online versus offline partner-seeking behaviours among dating app users.

Overall, the available evidence does not support a consistent or robust difference between online- and offline-oriented dating app users. Given the extreme heterogeneity and limited number of studies, findings should be interpreted with considerable caution (Figure 7).

#### Sexual intercourse experience by sex

Five studies contributed data to the meta-analysis examining sex-based differences in experience of sexual intercourse among dating app users, comprising 1081 women and 976 men. Across these studies, 662 women and 576 men reported having experienced sexual intercourse, as defined in the original reports.

Using a random-effects Mantel–Haenszel model, the pooled analysis indicated no statistically significant difference between women and men in the likelihood of reporting sexual intercourse experience (OR = 1.34, 95% CI, 0.48–3.75; Z = 0.56, *P* = .57). Although the point estimate suggested slightly higher odds among women, the CI was wide and included the null value, indicating substantial uncertainty around the pooled effect.

Between-study heterogeneity was very high (τ^2^ = 1.29; χ^2^ = 88.50, df = 4, *P* < .00001; *I*^2^ = 95%), reflecting pronounced variability in effect estimates across studies. This heterogeneity likely arises from differences in age composition of samples, cultural norms surrounding sexual debut, variation in definitions of sexual intercourse (eg, lifetime experience versus recent activity), and differences in study settings and recruitment strategies.

Inspection of the forest plot revealed considerable dispersion in study-specific ORs, with some studies reporting higher prevalence among women and others among men. No single study dominated the pooled estimate, but the wide CI indicates limited precision.

**Figure 8:**
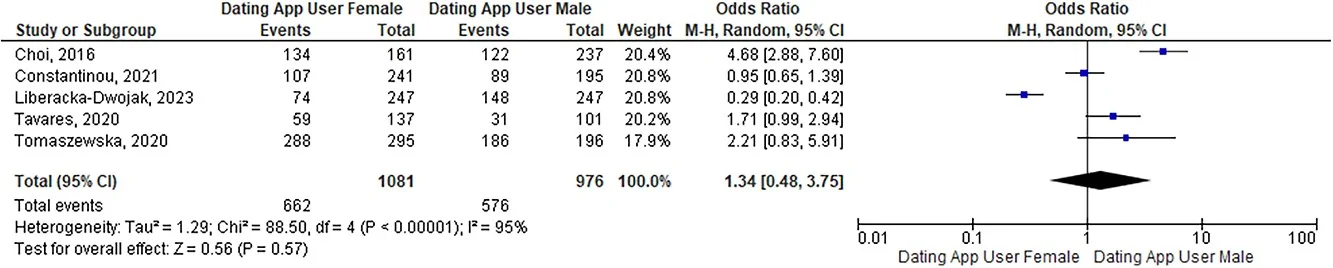
Sex-based differences in sexual intercourse experience among dating app users.

Overall, the available evidence does not support a consistent or robust sex-based difference in sexual intercourse experience among dating app users (Figure 8).

#### Sensitivity analysis

To assess the robustness of the pooled estimates and explore the source of the substantial between-study heterogeneity, leave-one-out sensitivity analyses were performed by sequentially excluding each study from the meta-analyses. The exclusion of individual studies did not materially alter the pooled effect estimates or substantially reduce the observed heterogeneity for any of the analyzed outcomes. These findings indicate that the high heterogeneity was not driven by any single study but rather reflects genuine methodological and clinical variability across the included studies, including differences in study populations, cultural contexts, outcome definitions, and measurement approaches.

#### Publication bias

Visual inspection of the funnel plot for the primary outcome (sex-based differences in dating app use) shows a moderate degree of asymmetry around the pooled effect estimate. While the majority of studies are distributed within the pseudo 95% confidence limits and cluster near the top of the funnel indicating relatively precise estimates from larger studies there is some dispersion among smaller studies, particularly on the left side of the plot.

This pattern suggests the possibility of small-study effects, whereby studies with smaller sample sizes report more extreme effect estimates. However, given the substantial between-study heterogeneity observed in the corresponding meta-analysis (*I*^2^ = 96%), asymmetry in the funnel plot is likely influenced not only by publication bias but also by true clinical and methodological heterogeneity, including differences in study populations, cultural contexts, recruitment settings, and operational definitions of dating app use.

**Figure 9:**
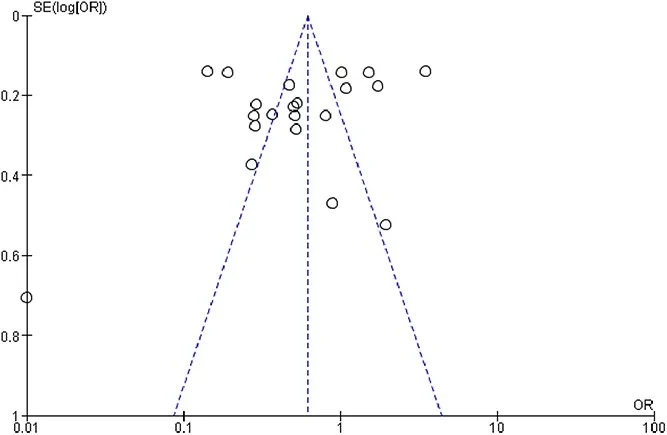
Funnel plot for sex-based differences in dating app use.

Importantly, the absence of a clear gap or truncation on one side of the funnel, together with the presence of several small studies reporting effects in both directions, indicates that strong publication bias cannot be conclusively inferred from visual inspection alone. In line with current methodological guidance, funnel plot findings were therefore interpreted cautiously and in conjunction with heterogeneity assessments and sensitivity analyses rather than as definitive evidence of reporting bias (Figure 9).

 Visual inspection of the funnel plot for the outcome sexual minority orientation (non-heterosexual identity) among dating app users suggests a moderate degree of asymmetry around the pooled effect estimate. While most studies cluster within the pseudo 95% confidence limits near the top of the funnel indicating relatively precise estimates from larger samples there is noticeable dispersion among smaller studies, particularly at lower levels of precision.

This asymmetry may reflect small-study effects, whereby studies with smaller sample sizes report more extreme ORs. However, given the substantial between-study heterogeneity observed in the corresponding meta-analysis (*I*^2^ = 92%), the funnel plot asymmetry is likely influenced not only by publication bias but also by true methodological and contextual heterogeneity. Potential contributors include variation in how sexual orientation was operationalized (identity-based vs behavior-based measures), cultural differences in disclosure of sexual minority status, and differences in the types of dating applications examined (general-population vs sexual-minority-focused platforms).

**Figure 10:**
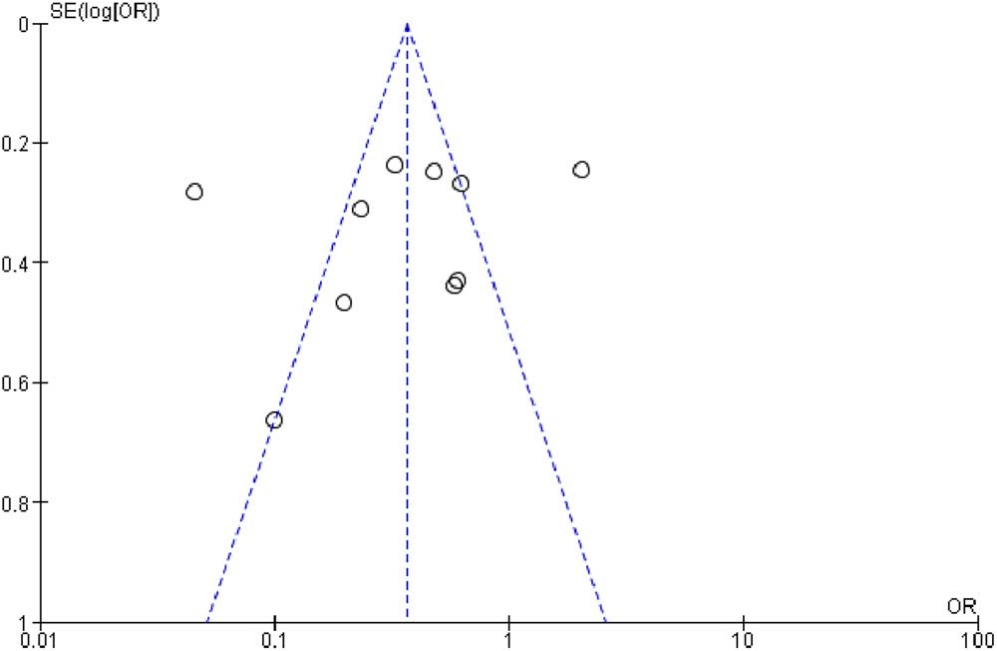
Funnel plot for sex-based differences in sexual orientation among dating app users.

Importantly, there is no clear evidence of systematic truncation or absence of studies on one side of the funnel, and smaller studies are present on both sides of the pooled effect estimate. Accordingly, strong publication bias cannot be conclusively inferred from visual inspection alone. Consistent with methodological guidance, funnel plot findings were therefore interpreted cautiously and considered alongside heterogeneity assessments rather than as definitive evidence of reporting bias (Figure 10).

#### Risk of bias and methodological quality assessment

The methodological quality and risk of bias of the included studies were assessed using the JBI Critical Appraisal Checklist for Analytical Cross-Sectional Studies. This tool was selected because all eligible studies employed observational, predominantly cross-sectional designs without experimental allocation of exposure.

The JBI checklist evaluates 8 key methodological domains relevant to bias in cross-sectional research: (1) appropriateness of the sampling frame, (2) adequacy of participant recruitment, (3) sample size considerations, (4) validity and reliability of exposure measurement (eg, dating app use), (5) validity and reliability of outcome measurement (eg, sexual behaviors, sexual health, or psychosocial outcomes), (6) identification of potential confounding factors, (7) strategies used to address confounding, and (8) appropriateness of statistical analyses. Each item was rated as “yes,” “no,” “unclear,” or “not applicable.” In line with JBI guidance, checklist items were not summed to generate an overall quality score. Instead, study-level judgements were derived qualitatively based on patterns across domains, with particular attention to participant selection and confounding control.

Risk-of-bias assessments indicated that the overall risk of bias across studies was low to moderate. Most studies demonstrated low risk in domains related to sampling frame, exposure assessment, and statistical analysis. The most common sources of potential bias were self-reported outcome measurement, which introduced some concerns regarding measurement validity, and incomplete control of confounding in a subset of studies. Only isolated instances of high risk were observed, primarily related to participant recruitment methods lacking adequate justification.

**Figure 11:**
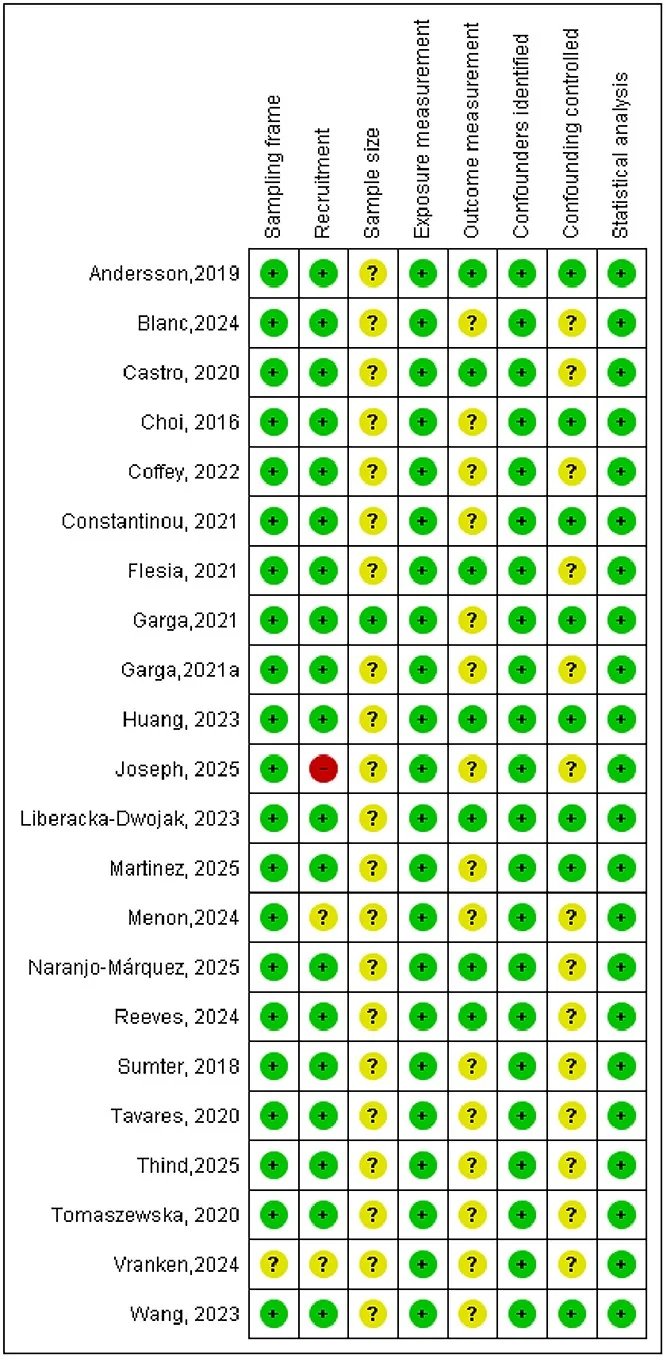
Traffic LAmp; Green indicates low risk of bias, yellow indicates some concerns, and red indicates high risk of bias.

To enhance transparency and facilitate comparability, JBI-derived judgements were summarized using traffic-light plots generated in RevMan, with green indicating low risk, yellow indicating some concerns, and red indicating high risk of bias. Review Manager was used solely for visual presentation and not as the appraisal instrument itself. Risk-of-bias assessments were not used as exclusion criteria but were considered when interpreting the findings and the robustness of the evidence (Table 2, Figure 11).

**Table 2:** Methodological quality and risk of bias assessment using the JBI checklist.

	**Study**	**1**	**2**	**3**	**4**	**5**	**6**	**7**	**8**
1	Andersson 2019	Yes	Yes	Unclear	Yes	Yes	Yes	Yes	Yes
2	Blanc 2024	Yes	Yes	Unclear	Yes	Unclear	Yes	Unclear	Yes
3	Castro 2020	Yes	Yes	Unclear	Yes	Yes	Yes	Unclear	Yes
4	Choi 2016	Yes	Yes	Unclear	Yes	Unclear	Yes	Yes	Yes
5	Coffey 2022	Yes	Yes	Unclear	Yes	Unclear	Yes	Unclear	Yes
6	Constantinou 2021	Yes	Yes	Unclear	Yes	Unclear	Yes	Yes	Yes
7	Flesia 2021	Yes	Yes	Unclear	Yes	Unclear	Yes	Yes	Yes
8	Garga 2021a (JMIR)	Yes	Yes	Yes	Yes	Unclear	Yes	Yes	Yes
9	Garga 2021 (HRJ)	Yes	Yes	Unclear	Yes	Unclear	Yes	Unclear	Yes
10	Huang 2023	Yes	Yes	Unclear	Yes	Yes	Yes	Yes	Yes
11	Joseph 2025	Yes	No	Unclear	Yes	Unclear	Yes	Unclear	Yes
12	Liberacka-Dwojak 2023	Yes	Yes	Unclear	Yes	Yes	Yes	Yes	Yes
13	Martinez 2025	Yes	Yes	Unclear	Yes	Unclear	Yes	Yes	Yes
14	Menon 2024	Yes	Unclear	Unclear	Yes	Unclear	Yes	Unclear	Yes
15	Reeves 2024	Yes	Yes	Unclear	Yes	Unclear	Yes	Yes	Yes
16	Sumter & Vandenbosch 2019	Yes	Yes	Unclear	Yes	Unclear	Yes	Unclear	Yes
17	Tavares 2020	Yes	Yes	Unclear	Yes	Unclear	Yes	Unclear	Yes
18	Thind 2025	Yes	Yes	Unclear	Yes	Unclear	Yes	Unclear	Yes
19	Tomaszewska 2020	Yes	Yes	Unclear	Yes	Unclear	Yes	Unclear	Yes
20	Wang 2023	Yes	Yes	Unclear	Yes	Unclear	Yes	Yes	Yes
21	Vranken 2024	Unclear	Unclear	Unclear	Yes	Unclear	Yes	Unclear	Unclear
22	Naranjo-Márquez 2025	Yes	Yes	Unclear	Yes	Unclear	Yes	Yes	Yes

## Discussion

The aim of this systematic review and meta-analysis was to synthesize sex-based differences in dating app use, related sexual behaviors, and sexual health outcomes using sex-stratified quantitative data from observational studies. The pooled findings suggest sex-based differences in several dating app–related behaviors and outcomes; however, these findings should be interpreted cautiously because substantial between-study heterogeneity was observed across most analyses. These findings highlight the value of sex-stratified evidence synthesis and may help inform future research and the development of tailored sexual health interventions in the context of digital dating.

Previous empirical studies and reviews have consistently reported sex differences in dating app use and related behaviors, with men more frequently reporting motivations related to casual sexual relationships and sexual exploration.^26–28^ In the present meta-analysis, the pooled estimates suggested that men were more likely than women to report dating app use. However, no statistically significant sex difference was observed for engagement in dating app–mediated casual sexual encounters, despite several individual studies reporting differences in this outcome. These findings are broadly consistent with previous research indicating that dating app motivations and behavioral patterns may differ by sex, although the magnitude and direction of these differences appear to vary across study populations. For example, Timmermans and Courtois (2018) reported that men more commonly used Tinder for short-term sexual interactions, whereas women demonstrated a more selective pattern of use.^26^ Similarly, Morlat et al. (2025) found that male users more frequently reported motivations related to casual sexual relationships and sexual exploration.^27^ These differences have been discussed in relation to gender norms and social expectations surrounding sexuality, which may influence both motivations for dating app use and behavioral outcomes.^28^ Likewise, a recent study among middle-aged adults also reported more intensive dating app use and a higher frequency of casual sexual encounters among men.^29^ Taken together, the existing literature suggests that gender norms and motivations may contribute to differences in dating app use. Nevertheless, the absence of a statistically robust pooled effect for casual sexual encounters, together with the substantial between-study heterogeneity observed in the present review, indicates that these findings should be interpreted with caution.

The literature increasingly emphasizes that the psychological and relational consequences of dating app use manifest differently depending on gender; these platforms are more strongly associated with mental well-being, body image, and relationship expectations, rather than behavioral outcomes, especially for female users.^2^^,^^9^ Taken together with previous literature, the present findings may suggest that psychological and relational aspects of dating app use could be particularly relevant for female users, although these outcomes were not directly synthesized in the present meta-analysis. The literature highlights that dating apps are not only a tool for finding partners for women, but also a social space closely related to body image, self-esteem, and relational expectations. Specifically, Chen and Lee (2024) found that dating app use showed significant correlations with body dissatisfaction, self-objectification, and mental well-being indicators in women.^2^ The increased pressure for evaluation and visibility among women on dating apps may contribute to the more pronounced psychological consequences. This indicates that female users experience app interactions not only through sexual behaviors but also through expectations of emotional and relational satisfaction. Coffey et al. (2022) reported that in individuals using dating apps for emotional connection or long-term relationships, repetitive casual sexual encounters may be associated with psychological tension and relationship dissatisfaction.^9^ Additionally, the lower motivation for short-term sexual relationships in women can increase psychological burden when app experiences don’t meet expectations. These observations should be interpreted cautiously and may reflect differences in motivations, expectations, and social norms rather than inherent behavioral differences. Therefore, the effects of dating apps on women are shaped more by psychological and relational processes than by behavioral outcomes.^2^^,^^9^

The high level of heterogeneity observed for many outcomes in this meta-analysis (*I*^2^ > 85%) necessitates a cautious approach to interpreting the findings. Significant methodological differences exist in the conceptualization of dating app use and associated outcomes within the current literature. Some studies treat dating app use as a general exposure variable, while others examine only casual sexual encounters or specific behavioral patterns occurring through the app.^28^ Similarly, outcomes such as sexual behaviors, sexual function, psychological outcomes, and STI history have been assessed using different measurement tools, definitions, and time periods.^2^

Another significant source of heterogeneity is the diversity in sample characteristics. The included studies encompass different age groups, cultural contexts, and sexual orientation distributions. In particular, sexual orientation and the type of dating app platform (apps for the general population vs. apps for specific sexual minorities) significantly influence the direction and magnitude of the results.^27^ Furthermore, the fact that all studies are observational and mostly cross-sectional in design limits causal inferences and leads to increased heterogeneity due to unmeasured confounders.^28^ In this context, the high heterogeneity does not necessarily invalidate the pooled analyses; however, it substantially limits the interpretability and generalizability of the pooled effect estimates. The observed variability likely reflects important methodological and contextual differences between studies rather than a single underlying effect. Therefore, the pooled effect estimates should be interpreted as average effects across heterogeneous study populations rather than as universal sex-based differences applicable to all contexts.

This cautious approach is consistent with broader evidence synthesis in digital health, where the reliability of pooled estimates depends not only on statistical significance but also on methodological consistency, heterogeneity, and the maturity of the available evidence base. Recent systematic reviews of emerging digital technologies have similarly emphasized cautious interpretation of pooled findings, particularly when evidence remains limited or heterogeneous.^30^

The current literature on sex-based differences in dating app use is largely limited to individual studies, lacking a holistic assessment across diverse behavioral and sexual health outcomes. This meta-analysis helps address this fragmented evidence base by quantitatively synthesizing sex-stratified data across multiple behavioral and health-related outcomes. Future evidence synthesis may also benefit from complementary research mapping and bibliometric approaches to identify emerging themes, methodological gaps, collaborative networks, and evolving research priorities within the rapidly expanding field of digital dating.^29^ Such approaches may facilitate greater harmonization of outcome definitions and improve comparability across future studies. If confirmed by future high-quality studies, these findings may help inform the development of tailored sexual and mental health interventions in the context of digital dating. However, given the substantial heterogeneity and the absence of statistically significant differences across several outcomes, these implications should be considered exploratory. More broadly, as with other emerging technology-mediated health interventions, translating research findings into practice requires careful consideration of implementation challenges, contextual variability, and the evolving nature of digital technologies.^31^

Another important limitation relates to the lack of harmonized outcome definitions across studies. Constructs such as dating app use, app-mediated casual sexual encounters, sexual function impairment, sexual minority orientation, STI history, and online versus offline partner-seeking behaviors were operationalized using different definitions, instruments, recall periods, and recruitment settings. This variability likely contributed to the substantial between-study heterogeneity observed across most analyses. Consequently, pooled estimates should be interpreted as reflecting broadly comparable rather than identical constructs.

Similar recommendations for cautious interpretation have been reported in recent meta-analyses synthesizing heterogeneous behavioral and preventive health evidence, where differences in outcome definitions and study populations substantially influenced pooled estimates.^32^

Although this review uses the term “sex differences” for consistency with the included studies, most primary studies classified participants using binary self-reported categories (women/men) without distinguishing biological sex from gender identity. Therefore, the findings should be interpreted within the limitations of the original studies.

## Conclusion

In conclusion, this systematic review and meta-analysis suggests that sex differences may exist across some domains of dating app use and related behavioral outcomes, particularly in patterns of app use, sexual function, and sexual orientation. However, statistically robust differences were not observed consistently across all analyzed outcomes, including casual sexual encounters, history of STIs, online versus offline partner-seeking, and sexual intercourse experience. However, substantial between-study heterogeneity indicates that these findings varied considerably across study contexts and should therefore be interpreted cautiously. These findings contribute to a more nuanced understanding of digital dating by suggesting that the health and relational implications of dating app use are contingent on sex-specific expectations, motivations, and social positioning. From a public health and research perspective, the results highlight the importance of incorporating sex-stratified analytical frameworks into future observational and interventional studies, as well as the development of tailored sexual and mental health strategies that reflect the differential experiences of women and men in digital dating contexts.

Overall, the pooled estimates should be interpreted as average effects across heterogeneous study populations rather than as universal sex differences. Future prospective studies using standardized definitions of dating app use and harmonized outcome measures are needed to clarify the magnitude and sources of sex-based variation. Replication in culturally diverse populations will also be important to determine whether the observed patterns are consistent across different settings.

## Supplementary Material

Supplementary_material_1_qfag079
