# Supplementary material for: Sex differences in digital dating app use and app-mediated sexual and health outcomes: a systematic review and meta-analysis
Source: Sex Med. 2026 Aug 24;14(5):qfag079. doi: 10.1093/sexmed/qfag079 (PMC13502903; doi:10.1093/sexmed/qfag079)
Supplement: Supplementary file 1 [file supplementary_material_1_qfag079.zip › Search_(2) (1).docx]

**PubMed/MEDLINE Search Strategy**

("Online Dating"[Mesh]

OR "dating app*"

OR "mobile dating"

OR "app-based dating"

OR Tinder

OR Bumble

OR Grindr)

AND

("Casual Sex"

OR "one-night stand*"

OR hookup*

OR "non-committed sexual relationship*"

OR "casual sexual encounter*")

AND

("Sexual Behavior"[Mesh]

OR "sexual intercourse"

OR "partner seeking"

OR "online dating behavior"

OR "offline dating behavior")

AND

("Sex Differences"[Mesh]

OR "gender difference*"

OR women

OR men)

OR ("Sexually Transmitted Diseases"[Mesh] OR STI* OR STD*)

**Web of Science (Core Collection) Search Strategy**

TS = (

("dating app*" OR "online dating" OR "mobile dating" OR "app-based dating"

OR Tinder OR Bumble OR Grindr)

AND

("casual sex" OR "one-night stand*" OR hookup* OR "non-committed sexual relationship*")

AND

("sexual behavior" OR "sexual intercourse" OR "partner seeking"

OR "online dating" OR "offline dating")

AND

("sex difference*" OR "gender difference*" OR women OR men)

)OR

TS = ("sexually transmitted infection*" OR STI* OR STD*)

**PsycINFO Search Strategy**

(DE "Online Dating"

OR "dating app*"

OR "mobile dating"

OR Tinder

OR Bumble

OR Grindr)

AND

("casual sex"

OR "one-night stand*"

OR hookup*

OR "non-committed sexual relationship*")

AND

("sexual behavior"

OR "sexual intercourse"

OR "partner seeking")

AND

("gender difference*"

OR "sex difference*"

OR women

OR men)

OR

("sexually transmitted infection*"

OR STI*

OR STD*)

**Scopus Search Strategy**

TITLE-ABS-KEY (

("dating app*" OR "online dating" OR "mobile dating" OR "app-based dating"

OR Tinder OR Bumble OR Grindr)

AND

("casual sex" OR "one-night stand*" OR hookup* OR "non-committed sexual relationship*")

AND

("sexual behavior" OR "sexual intercourse" OR "partner seeking"

OR "online dating" OR "offline dating")

AND

("sex difference*" OR "gender difference*" OR women OR men)

)

OR

TITLE-ABS-KEY ("sexually transmitted infection*" OR STI* OR STD*)

**Cochrane Library Search Strategy**

("online dating"

OR "dating app*"

OR Tinder

OR Bumble

OR Grindr)

AND

("casual sex"

OR "one-night stand*"

OR hookup*)

AND

("sexual behavior"

OR "sexual intercourse")

AND

(women OR men OR "sex difference*" OR "gender difference*")
