# Supplementary material for: Sex differences in digital dating app use and app-mediated sexual and health outcomes: a systematic review and meta-analysis
Source: Sex Med. 2026 Aug 24;14(5):qfag079. doi: 10.1093/sexmed/qfag079 (PMC13502903; doi:10.1093/sexmed/qfag079)
Supplement: Supplementary file 1 [file supplementary_material_1_qfag079.zip › excluded_manuscipt_(1)_SM-26-0507.R1.docx]

1. Tran, A., Darboe, M., Goyal, A. *et al.* Association between dating app use and unhealthy weight control behaviors and muscle enhancing behaviors in sexual minority men: a cross-sectional study. *BMC Public Health* **23**, 838 (2023). <https://doi.org/10.1186/s12889-023-15715-7>
2. Efendi, Z., Al Firdha Hanim, M., Abdullah, I., Jubba, H., & Nurazizah, S. (2025). The influence of dating apps on sexual behavior among female students: A sociocultural analysis. **Masyarakat, Kebudayaan dan Politik, 38**(3), 262–275. <https://doi.org/10.20473/mkp.V38I32025.262-275>
3. Rosenberg M, Luetke M, Hensel D, Kianersi S, Fu TC, Herbenick D. Depression and loneliness during April 2020 COVID-19 restrictions in the United States, and their associations with frequency of social and sexual connections. Soc Psychiatry Psychiatr Epidemiol. 2021 Jul;56(7):1221-1232. doi: 10.1007/s00127-020-02002-8. Epub 2021 Jan 2. PMID: 33386873; PMCID: PMC7778397.-- Dating apps are not focused on primary exposure, nor on sexual risk.
4. Wong, W. C. W., Song, L., See, C., Lau, S. T. H., Sun, W. H., Choi, K. W. Y., & Tucker, J. (2020). Using Crowdsourcing to Develop a Peer-Led Intervention for Safer Dating App Use: Pilot Study. JMIR formative research, 4(4), e12098. https://doi.org/10.2196/12098- Excluded: qualitative / formative intervention development study; no quantitative association between dating app use and sexual behavior outcomes.
5. Newlands RT, Denning DM, Massey KS, Benuto LT. Safe Dating in the Digital Era: Protective Behavioral Strategies in Dating Behaviors Facilitated by Dating Applications. Violence Vict. 2022 Apr 1;37(2):185-200. doi: 10.1891/VV-D-19-00173. PMID: 35354656. Excluded from quantitative synthesis: outcomes focused on protective behavioral strategies rather than sexual risk or sexual health outcomes.
6. Beikzadeh M, Holloway IW, Kärkkäinen K, Hong C, Cascalheira C, Wu ESC, BokaC, Avendaño AC, Yonko EA, Sarrafzadeh M. Identifying Substance Use and High-Risk Sexual Behavior Among Sexual and Gender Minority Youth by Using Mobile Phone Data: Development and Validation Study. Online J Public Health Inform. 2025 Au 12;17:e68013. doi: 10.2196/68013. PMID: 40795338; PMCID: PMC12360732.- Excluded from quantitative synthesis: study focused on machine-learning–based prediction of high-risk sexual behaviors using mobile phone data rather than examining associations between dating app use and sexual or health outcomes.
7. Perez J, Pepping CA. Relationship experiences of transgender and non-binary adults: Exploring dating goals, relationship structures, minority stress, sexual fetishization, and relationship victimization. Int J Transgend Health. 2024 Mar 4;26(4):1026-1045. doi: 10.1080/26895269.2024.2316690. PMID: 41180926; PMCID:PMC12573564.- Excluded from quantitative synthesis: study focused on relationship experiences, sexual fetishization, and minority stress among transgender and non-binary adults without examining dating app use as a primary exposure or reporting extractable sexual health or behavioral outcomes.
8. Sheoran, B., Silva, C. L., Lykens, J. E., Gamedze, L., Williams, S., Ford, J. V., & Habel, M. A. (2016). YTH StreetConnect: Development and Usability of a Mobile App for Homeless and Unstably Housed Youth. JMIR mHealth and uHealth, 4(3), e82. https://doi.org/10.2196/mhealth.5168-Excluded because the study focused on the development and usability of a service-navigation mobile health application rather than dating app use or its association with sexual, psychological, or behavioral health outcomes.
9. Halary, S., Daubois, L., Terrat, Y., Ellenberger, S., Wöstemeyer, J., & Hijri, M. (2013). Mating type gene homologues and putative sex pheromone-sensing pathway in arbuscular mycorrhizal fungi, a presumably asexual plant root symbiont. PloS one, 8(11), e80729. https://doi.org/10.1371/journal.pone.0080729-Excluded because the study focused on molecular and genetic mechanisms of sexual reproduction in fungi and did not involve human participants, dating app use, or sexual or behavioral health outcomes.
10. Kiekens, W. J., Baams, L., Fish, J. N., & Watson, R. J. (2022). Associations of Relationship Experiences, Dating Violence, Sexual Harassment, and Assault With Alcohol Use Among Sexual and Gender Minority Adolescents. *Journal of interpersonal violence*, *37*(17-18), NP15176–NP15204. https://doi.org/10.1177/08862605211001469-Excluded because the study examined offline relationship experiences, dating violence, and alcohol use among sexual and gender minority adolescents and did not assess dating app or online dating platform use.
11. Eisman, A. B., Hicks, M., Kernsmith, P. D., Rupp, L., Smith-Darden, J. P., & Zimmerman, M. A. (2021). Adapting an evidence-based positive youth development intervention to prevent sexual and teen dating violence. *Translational behavioral medicine*, *11*(1), 74–86. <https://doi.org/10.1093/tbm/ibz156>-Excluded because the study focused on an offline, school- and community-based intervention to prevent teen dating violence and did not assess dating app or online dating platform use.
12. Kim, K., Moss, C., Park, J. J., & Wekerle, C. (2022). Child Maltreatment and the Child Welfare System as Environmental Factors in the International Classification of Functioning. *Frontiers in rehabilitation sciences*, *2*, 710629. https://doi.org/10.3389/fresc.2021.710629-Excluded because the study focused on child maltreatment and the child welfare system within the ICF framework and did not examine dating app use, online dating contexts, or sexual or psychosocial outcomes related to digital dating.
13. Meiksin, R., Bonell, C., Bhatia, A., Melendez-Torres, G. J., Kyegombe, N., & Kohli, A. (2024). Social Norms About Dating and Relationship Violence and Gender Among Adolescents: Systematic Review of Measures Used in Dating and Relationship Violence Research. *Trauma, violence & abuse*, *25*(1), 448–462. https://doi.org/10.1177/15248380231155526
